# Supplementary material for: Neoadjuvant chemotherapy efficacy and prognosis in HER2-low and HER2-zero breast cancer patients by HR status: a retrospective study in China
Source: PeerJ. 2024 May 29;12:e17492. doi: 10.7717/peerj.17492 (PMC11143972; doi:10.7717/peerj.17492)
Supplement: Supplemental Information 1 [file peerj-12-17492-s001.docx]

age：1= “≤40”；2= “41-49”；3= “50-74”；4= “≥75”

Menstrual status：0= “premenopausal”；1= “postmenopausal”

T stage：1= “T1”；2= “T2”；3= “T3”；4= “T4”

N stage：1= “N0”；2= “N1-3”；3= “unknown”

pathological type：1= “invasive ductal carcinoma”；2= “invasive lobular carcinoma”；3= “other”

histological grade：1= “Ⅰ-Ⅱ”；2= “Ⅲ”；3= “unknown”

HR：1= “negative”；2= “positive”

ER：1= “negative”；2= “positive”

PR：1= “negative”；2= “positive”

HER2_2：1= “HER2-0”；2= “HER2-low”

HER2_3：1= “HER2-0”；2= “HER2-1+”；3= “HER2-2+”

ki67：1= “≤14%”；2= “＞14%”

pCR：0= “non-pCR”；1= “pCR”
