# Supplementary material for: Neoadjuvant chemotherapy efficacy and prognosis in HER2-low and HER2-zero breast cancer patients by HR status: a retrospective study in China
Source: PeerJ. 2024 May 29;12:e17492. doi: 10.7717/peerj.17492 (PMC11143972; doi:10.7717/peerj.17492)
Supplement: Supplemental Information 3 [file peerj-12-17492-s003.docx]

STROBE Statement—checklist of items that should be included in reports of observational studies

|  | Item No. | Recommendation | Page  No. | Relevant text from manuscript |
| --- | --- | --- | --- | --- |
| **Title and abstract** | 1 | (*a*) Indicate the study’s design with a commonly used term in the title or the abstract | 1 | a case-control study |
|  |  | (*b*) Provide in the abstract an informative and balanced summary of what was done and what was found | 2 | . |
| Introduction | | | |  |
| Background/rationale | 2 | Explain the scientific background and rationale for the investigation being reported | 3 | HER2-negative cases may be either Luminal or TNBC and are treated according to these classifications; however, this is both imprecise and insufficient. |
| Objectives | 3 | State specific objectives, including any prespecified hypotheses | 3 | the objective of this study was to evaluate the pathological complete response (pCR) and disease-free survival (DFS) in HER2-low patients |
| Methods | | | |  |
| Study design | 4 | Present key elements of study design early in the paper | 4 | study design |
| Setting | 5 | Describe the setting, locations, and relevant dates, including periods of recruitment, exposure, follow-up, and data collection | 4;  6 | Collection of patient data;  Follow-up |
| Participants | 6 | (*a*) *Cohort study*—Give the eligibility criteria, and the sources and methods of selection of participants. Describe methods of follow-up  *Case-control study*—Give the eligibility criteria, and the sources and methods of case ascertainment and control selection. Give the rationale for the choice of cases and controls  *Cross-sectional study*—Give the eligibility criteria, and the sources and methods of selection of participants | 6 | pCR was defined as the absence of invasive carcinoma in the breast or axillary lymph nodes after chemotherapy despite the possible presence of in situ residual ductal carcinoma components within the breast lesion (ypT0/is ypN0). |
|  |  | (*b*) *Cohort study*—For matched studies, give matching criteria and number of exposed and unexposed  *Case-control study*—For matched studies, give matching criteria and the number of controls per case |  | none |
| Variables | 7 | Clearly define all outcomes, exposures, predictors, potential confounders, and effect modifiers. Give diagnostic criteria, if applicable | 6;  4;  5. | Neoadjuvant treatment and efficacy evaluation;  The data included information on age; menopausal status; clinical stage (T and N); pathological type; histological grade; hormone receptor (HR); estrogen receptor (ER); progesterone receptor (PR); HER2 status; Ki-67; neoadjuvant chemotherapy regimen and efficacy;  Diagnosis, IHC and staging system. |
| Data sources/ measurement | 8* | For each variable of interest, give sources of data and details of methods of assessment (measurement). Describe comparability of assessment methods if there is more than one group | 5 | Diagnosis, IHC and staging system |
| Bias | 9 | Describe any efforts to address potential sources of bias | 5 | Every semiquantitative scoring of immunostained section was analyzed by two independent pathologists. The sample was reassessed in case of discordance. |
| Study size | 10 | Explain how the study size was arrived at | 4 | Sample size |

Continued on next page

| Quantitative variables | 11 | Explain how quantitative variables were handled in the analyses. If applicable, describe which groupings were chosen and why | 5 | Diagnosis, IHC and staging system |
| --- | --- | --- | --- | --- |
| Statistical methods | 12 | (*a*) Describe all statistical methods, including those used to control for confounding | 6 | Statistical analysis |
|  |  | (*b*) Describe any methods used to examine subgroups and interactions | 5 | We further conducted stratified research based on the hormone receptor (HR) status. |
|  |  | (*c*) Explain how missing data were addressed | 6 | A total of 99 cases were excluded in survival analysis due to missing data on survival state and survival time |
|  |  | (*d*) *Cohort study*—If applicable, explain how loss to follow-up was addressed  *Case-control study*—If applicable, explain how matching of cases and controls was addressed  *Cross-sectional study*—If applicable, describe analytical methods taking account of sampling strategy |  | none |
|  |  | (*e*) Describe any sensitivity analyses |  | none |
| Results | | | | |
| Participants | 13* | (a) Report numbers of individuals at each stage of study—eg numbers potentially eligible, examined for eligibility, confirmed eligible, included in the study, completing follow-up, and analysed | 5 | A study flow diagram is shown in in Figure 1 |
|  |  | (b) Give reasons for non-participation at each stage | 5 |  |
|  |  | (c) Consider use of a flow diagram | 5 | Figure 1 |
| Descriptive data | 14* | (a) Give characteristics of study participants (eg demographic, clinical, social) and information on exposures and potential confounders | 7 | Table 1 Comparison of clinicopathological factors between HER2-low and HER2-zero breast cancer patients |
|  |  | (b) Indicate number of participants with missing data for each variable of interest | 7 | Table 1 |
|  |  | (c) *Cohort study*—Summarise follow-up time (eg, average and total amount) |  |  |
| Outcome data | 15* | *Cohort study*—Report numbers of outcome events or summary measures over time |  |  |
|  |  | *Case-control study—*Report numbers in each exposure category, or summary measures of exposure | 8 | Table 3 Correlation between factors and pCR rate in HER2-negative breast cancer patients |
|  |  | *Cross-sectional study—*Report numbers of outcome events or summary measures |  |  |
| Main results | 16 | (*a*) Give unadjusted estimates and, if applicable, confounder-adjusted estimates and their precision (eg, 95% confidence interval). Make clear which confounders were adjusted for and why they were included | 8 | Variables found to be significant (p < 0.05) were included in the binary logistic regression analysis. |
|  |  | (*b*) Report category boundaries when continuous variables were categorized | 5 | According to the St. Gallen Guideline 2013, Ki-67 expression is classified as high or low based on a cutoff of 14% |
|  |  | (*c*) If relevant, consider translating estimates of relative risk into absolute risk for a meaningful time period |  | none |

Continued on next page

| Other analyses | 17 | Report other analyses done—eg analyses of subgroups and interactions, and sensitivity analyses | 11 | Therefore, subgroup analysis of pCR rate was further conducted according to HR status. |
| --- | --- | --- | --- | --- |
| Discussion | | | | |
| Key results | 18 | Summarise key results with reference to study objectives | 13 | Conclusions |
| Limitations | 19 | Discuss limitations of the study, taking into account sources of potential bias or imprecision. Discuss both direction and magnitude of any potential bias | 13 | Limitations |
| Interpretation | 20 | Give a cautious overall interpretation of results considering objectives, limitations, multiplicity of analyses, results from similar studies, and other relevant evidence | 13 | It is worth noting that the current studies, including our own research, have a median follow-up period of approximately 5 years. Longer-term monitoring is necessary to fully assess the prognosis of breast cancer. |
| Generalisability | 21 | Discuss the generalisability (external validity) of the study results | 13 | It was a single-center retrospective study, necessitating further verification of its extrapolation |
| Other information | |  | | |
| Funding | 22 | Give the source of funding and the role of the funders for the present study and, if applicable, for the original study on which the present article is based | 14 | Funding |

*Give information separately for cases and controls in case-control studies and, if applicable, for exposed and unexposed groups in cohort and cross-sectional studies.

**Note:** An Explanation and Elaboration article discusses each checklist item and gives methodological background and published examples of transparent reporting. The STROBE checklist is best used in conjunction with this article (freely available on the Web sites of PLoS Medicine at http://www.plosmedicine.org/, Annals of Internal Medicine at http://www.annals.org/, and Epidemiology at http://www.epidem.com/). Information on the STROBE Initiative is available at www.strobe-statement.org.
