# Supplementary material for: Neoadjuvant chemotherapy efficacy and prognosis in HER2-low and HER2-zero breast cancer patients by HR status: a retrospective study in China
Source: PeerJ. 2024 May 29;12:e17492. doi: 10.7717/peerj.17492 (PMC11143972; doi:10.7717/peerj.17492)
Supplement: Supplemental Information 4 — HER2, human epidermal growth factor receptor 2; ER, Estrogen receptor; pCR, pathological complete response; OR, odd ratio; CI, confidence intervals. [file peerj-12-17492-s004.docx]

**Table S1.** Factors associated with pCR rate in ER-positive and ER-negative subgroups

| Factors | ER-positive | | | | | | | ER-negative | | | | |
| --- | --- | --- | --- | --- | --- | --- | --- | --- | --- | --- | --- | --- |
|  |  | pCR  (n=69) | Univariate Analysis | | Multivariate  Analysis | |  | | pCR  (n=121) | Univariate Analysis | Multivariate  Analysis | |
|  |  |  | *p*-value | OR (95%CI) | | *p*-value |  |  |  | *p*-value | OR (95%CI) | *p*-value |
| Age |  |  | 0.918 |  | |  |  | |  | 0.052 |  |  |
| ≤40 | 127 | 14 (11.0%) |  |  | |  | 67 | | 19 (28.4%) |  |  |  |
| 41–49 | 191 | 20 (10.5%) |  |  | |  | 92 | | 31 (33.7%) |  |  |  |
| 50–74 | 334 | 35 (10.5%) |  |  | |  | 158 | | 71 (44.9%) |  |  |  |
| ≥75 | 4 | 0 (0.0%) |  |  | |  | 2 | | 0 (0.0%) |  | - | - |
| Menstrual status |  |  | 0.844 |  | |  |  | |  | 0.261 |  |  |
| premenopausal | 259 | 28 (10.8%) |  |  | |  | 171 | | 60 (35.1%) |  |  |  |
| postmenopausal | 397 | 41 (10.3%) |  |  | |  | 148 | | 61 (41.2%) |  |  |  |
| T stage |  |  | <0.001 |  | |  |  | |  | <0.001 |  |  |
| T1 | 74 | 17 (23.0%) |  | ref | |  | 88 | | 50 (56.8%) |  | ref |  |
| T2 | 339 | 42 (12.4%) |  | 0.60 (0.28–1.30) | | 0.197 | 157 | | 54 (34.4%) |  | 0.41 (0.22–0.78) | 0.006 |
| T3 | 186 | 7 (3.8%) |  | 0.13 (0.05–0.40) | | <0.001 | 54 | | 12 (22.2%) |  | 0.26 (0.11–0.63) | 0.003 |
| T4 | 57 | 3 (5.3%) |  | 0.28 (0.07–1.15) | | 0.078 | 20 | | 5 (25.0%) |  | 0.39 (0.11–1.35) | 0.136 |
| N stage |  |  | <0.001 |  | |  |  | |  | 0.012 |  |  |
| N0 | 239 | 58 (24.3%) |  | ref | |  | 132 | | 60 (45.5%) |  | ref |  |
| N1–3 | 401 | 11 (2.7%) |  | 0.05 (0.02–0.12) | | <0.001 | 184 | | 58 (31.5%) |  | 0.65 (0.37–1.13) | 0.128 |
| unknown | 16 | 0 (0.0%) |  | - | | - | 3 | | 3 (100.0%) |  | - | - |
| Pathological type |  |  | 0.425 |  | |  |  | |  | 0.280 |  |  |
| Invasive ductal carcinoma | 585 | 61 (10.4%) |  |  | |  | 295 | | 115 (39.0%) |  |  |  |
| Invasive lobular carcinoma | 45 | 3 (6.7%) |  |  | |  | 2 | | 0 (0.0%) |  |  |  |
| Others | 26 | 5 (19.2%) |  |  | |  | 22 | | 6 (27.3%) |  |  |  |
| Histological grade |  |  | <0.001 |  | |  |  | |  | <0.001 |  |  |
| Ⅰ-Ⅱ | 394 | 28 (7.1%) |  | ref | |  | 141 | | 30 (21.3%) |  | ref |  |
| Ⅲ | 262 | 41 (15.6%) |  | 1.98 (0.98–1.02) | | 0.059 | 153 | | 84 (54.9%) |  | 4.45 (2.57–7.70) | <0.001 |
| unknown | 0 | 0 (0.0%) |  | - | | - | 25 | | 7 (28%) |  | 1.44 (0.55–3.76) | 0.932 |
| Ki-67 |  |  | 0.279 |  | |  |  | |  | 0.136 |  |  |
| ≤14% | 106 | 8 (7.5%) |  |  | |  | 22 | | 5 (22.7%) |  |  |  |
| ＞14% | 550 | 61 (11.1%) |  |  | |  | 297 | | 116 (39.1%) |  |  |  |
| HER2 status |  |  | 0.008 |  | |  |  | |  | 0.943 |  |  |
| zero | 237 | 35 (14.8%) |  | ref | |  | 159 | | 60 (37.7%) |  |  |  |
| low | 419 | 34 (8.1%) |  | 0.58 (0.30–0.95) | | 0.048 | 160 | | 61 (38.1%) |  |  |  |

HER2, human epidermal growth factor receptor 2; ER, Estrogen receptor; pCR, pathological complete response; OR, odd ratio; CI, confidence intervals.
