# Supplementary material for: Neoadjuvant chemotherapy efficacy and prognosis in HER2-low and HER2-zero breast cancer patients by HR status: a retrospective study in China
Source: PeerJ. 2024 May 29;12:e17492. doi: 10.7717/peerj.17492 (PMC11143972; doi:10.7717/peerj.17492)
Supplement: Supplemental Information 5 — HER2, human epidermal growth factor receptor 2; PR, progesterone receptor; pCR, pathological complete response; OR, odd ratio; CI, confidence intervals. [file peerj-12-17492-s005.docx]

**Table S2.** Factors associated with pCR rate in PR-positive and PR-negative subgroups

| Factors | PR-positive | | | | | | | PR-negative | | | | |
| --- | --- | --- | --- | --- | --- | --- | --- | --- | --- | --- | --- | --- |
|  |  | pCR  (n=53) | Univariate Analysis | | Multivariate  Analysis | |  | | pCR  (n=137) | Univariate Analysis | Multivariate  Analysis | |
|  |  |  | *p*-value | OR (95%CI) | | *p*-value |  |  |  | *p*-value | OR (95%CI) | *p*-value |
| Age |  |  | 0.361 |  | |  |  | |  | 0.095 |  |  |
| ≤40 | 121 | 14 (11.6%) |  |  | |  | 73 | | 19 (26.0%) |  |  |  |
| 41–49 | 179 | 15 (8.4%) |  |  | |  | 104 | | 36 (34.6%) |  |  |  |
| 50–74 | 291 | 24 (8.2%) |  |  | |  | 201 | | 82 (40.8%) |  |  |  |
| ≥75 | 4 | 0 (0.0%) |  |  | |  | 2 | | 0 (0.0%) |  |  |  |
| Menstrual status |  |  | 0.731 |  | |  |  | |  | 0.717 |  |  |
| premenopausal | 245 | 23 (9.4%) |  |  | |  | 185 | | 65 (35.1%) |  |  |  |
| postmenopausal | 350 | 30 (8.6%) |  |  | |  | 195 | | 72 (36.9%) |  |  |  |
| T stage |  |  | <0.001 |  | |  |  | |  | <0.001 |  |  |
| T1 | 67 | 13 (19.4%) |  | ref | |  | 95 | | 54 (56.8%) |  | ref |  |
| T2 | 312 | 32 (10.1%) |  | 0.55 (0.26–1.17) | | 0.119 | 184 | | 64 (34.8%) |  | 0.42 (0.23–0.77) | 0.005 |
| T3 | 163 | 5 (3.1%) |  | 0.16 (0.05–0.47) | | <0.001 | 77 | | 14 (18.2%) |  | 0.22 (0.10–0.49) | <0.001 |
| T4 | 53 | 3 (5.7%) |  | 0.30 (0.08–1.15) | | 0.078 | 24 | | 5 (20.8%) |  | 0.31 (0.09–1.02) | 0.054 |
| N stage |  |  | 0.984 |  | |  |  | |  | 0.007 |  |  |
| N0 | 223 | 53 (23.8%) |  |  | |  | 148 | | 65 (43.9%) |  | ref |  |
| N1–3 | 356 | 0 (0.0%) |  |  | |  | 229 | | 69 (30.1%) |  | 0.62 (0.37–1.04) | 0.070 |
| unknown | 16 | 0 (0.0%) |  | - | | - | 3 | | 3 (100.0%) |  | - | - |
| Pathological type |  |  | 0.398 |  | |  |  | |  | 0.177 |  |  |
| Invasive ductal carcinoma | 532 | 47 (8.8%) |  |  | |  | 348 | | 129 (37.1%) |  |  |  |
| Invasive lobular carcinoma | 42 | 3 (7.1%) |  |  | |  | 5 | | 0 (0.0%) |  |  |  |
| Others | 21 | 3 (14.3%) |  |  | |  | 27 | | 8 (29.7%) |  |  |  |
| Histological grade |  |  | 0.036 |  | |  |  | |  | <0.001 |  |  |
| Ⅰ–Ⅱ | 372 | 26 (7.0%) |  | ref | |  | 163 | | 32 (19.6%) |  | ref |  |
| Ⅲ | 223 | 27 (12.1%) |  | 1.69 (0.95–3.03) | | 0.075 | 192 | | 98 (51.0%) |  | 4.73 (2.81–7.98) | <0.001 |
| unknown | 0 | 0 (0.0%) |  | - | | - | 25 | | 7 (28.0%) |  | 1.06 (0.37–3.03) | 0.912 |
| Ki-67 |  |  | 0.703 |  | |  |  | |  | 0.049 |  |  |
| ≤14% | 101 | 8 (7.9%) |  |  | |  | 27 | | 5 (18.5%) |  | ref |  |
| ＞14% | 494 | 45 (9.1%) |  |  | |  | 353 | | 132 (37.4%) |  | 3.84 (1.25–11.79) | 0.018 |
| HER2 status |  |  | 0.423 |  | |  |  | |  | 0.240 |  |  |
| zero | 206 | 21 (10.2%) |  |  | |  | 190 | | 74 (38.9%) |  |  |  |
| low | 389 | 32 (8.2%) |  |  | |  | 190 | | 63 (33.2%) |  |  |  |

HER2, human epidermal growth factor receptor 2; PR, progesterone receptor; pCR, pathological complete response; OR, odd ratio; CI, confidence intervals.
